# Statins and Abnormal Liver Function Tests: Is There a Correlation?

**DOI:** 10.7759/cureus.10145

**Published:** 2020-08-30

**Authors:** Jibran Ashraf, M Ali Khan, Syed Minhaj, Shahzad Khatti, Khawaja M Aarij, Muhammad Shehzad, Tariq M Khan

**Affiliations:** 1 Cardiology, National Institute of Cardiovascular Diseases, Karachi, PAK; 2 Gastroenterology, Jinnah Postgraduate Medical Centre, Karachi, PAK; 3 Cardiac Surgery, National Institute of Cardiovascular Diseases, Karachi, PAK; 4 Interventional Cardiology, National Institute of Cardiovascular Diseases, Karachi, PAK; 5 Noninvasive Imaging, National Institute of Cardiovascular Diseases, Karachi, PAK

**Keywords:** statin, correlation, liver function tests, lft

## Abstract

Background

Statins or 3-hydroxy-3-methyl-glutaryl-coenzyme A (HMG-CoA) reductase inhibitors are one of the most commonly prescribed medications in cardiac patients. Just like any other class of drugs, they have the potential to cause liver injury over time even with judicious use. This drug-induced liver injury (DILI) can be either direct (hepatocellular) or idiosyncratic. As with multiple other hepatic pathologies, DILI may be asymptomatic or clinically silent. Therefore, it is prudent to carry out liver function tests (LFTs) from time to time. LFTs are an inexpensive, noninvasive, and quick first-line investigation to monitor liver status. However, the pattern of liver injury with statin use is not specific and a correlation over time may not be apparent.

Aims

To evaluate derangement in LFTs over time with respect to statin use and determine if a correlation exists.

Methods

This was a retrospective observational cohort. All data were collected from the online database of the National Institute of Cardiovascular Diseases (NICVD), Karachi. Patients admitted to the NICVD from July 1, 2018, to December 31, 2018, were eligible for inclusion in the study. Only patients already taking a statin (in any dose) were considered for inclusion. LFTs were recorded from the database at inclusion, post-induction at six and 12 months. Extensive workup was done and great care taken to rule out other diseases that may have affected the LFTs.

Results

Two hundred and four patients were eventually inducted into the study after a meticulous exclusion process. The male to female ratio was 4:1. The mean duration of statin use before induction into the study was 19.92±14.34 months. Patients were predominantly using only one of two statins, i.e., rosuvastatin 20mg/day or atorvastatin 40 mg/day. Elevations of LFTs were seen with both drugs throughout the study period. These elevations were almost always <2x the upper limit of normal (ULN); greater elevations were seen with atorvastatin 40 mg/day. The derangement in LFTs persisted and improvement was not seen.

Conclusions

Statins cause dose-dependent borderline elevations of liver function tests over time. These elevations are clinically and statistically insignificant and should not deter physicians from prescribing or continuing statins.

## Introduction

Statins or 3-hydroxy-3-methyl-glutaryl-coenzyme A (HMG-CoA) reductase inhibitors reduce the production of cholesterol precursors and thereby decrease overall cholesterol biosynthesis [1]. Statins reduce low-density lipid-cholesterol (LDL-C) and increase high-density lipid-cholesterol levels (HDL-C) [2]. Statins also have additional so-called "pleiotropic" pharmacodynamic effects [3]. These "pleiotropic" effects include reduced endothelial dysfunction and increased nitrous oxide bioavailability, inhibition of inflammatory processes, stabilization of atherosclerotic plaques, and antioxidant properties. At the end of the day, statins reduce the atherogenic lipoprotein burden and have been shown to reduce the risk and incidence of nonfatal myocardial infarction, ischemic stroke, and all-cause cardiovascular mortality [4]. In the setting of secondary prevention, statins, especially rosuvastatin, reduce intima thickness and the need for revascularization therapy, leading to improved survival rates [5]. All these aspects make statins ideal drugs for the treatment and prevention of cardiovascular diseases. 

Statins have been an overwhelming success since their introduction in the 1980s and went on to become one of the top-selling medications in the world [6]. Statins have few and very rare side effects [7] but with such precipitous use, myth and reality tend to overlap [8]. One such fact or legend is that of abnormal liver function tests (LFTs). This could be due to the fact that like most medications, statins are primarily metabolized in the liver [9]; this can lead to direct hepatocellular injury, causing deranged liver function tests.

Secondly, virtually any drug has the potential to cause an idiosyncratic liver injury termed as ‘drug-induced liver injury’ or ‘DILI’ that may be hepatocellular, cholestatic, or mixed pattern in nature [10], statins are no exception to this phenomenon. Liver toxicity has hardly ever been reported but mild elevations of alanine aminotransferase (ALT) or aspartate aminotransferase (AST) have been recorded [11]. Conversely, in patients with liver diseases, statins have actually led to improved levels of ALT and/or AST [12]. Thus, the causal relationship between statins and LFTs remains a complicated one.

Where better to gain further insight into this relationship than a cardiac care unit. Here, most patients are already taking statins and even more are being freshly prescribed one. The old adage “once on a statin, always on a statin” holds true in this setting; almost all patients are prescribed a statin for life or until the next adverse event. Patients are highly compliant owing to the seriousness of cardiac illnesses and maintain follow-up. Therefore variables such as duration of use, dosage, type of statin (hydrophilic or lipophilic), and side effects can be easily monitored over time.

This was, by no means, groundbreaking research. Whatever little data that was available from Pakistan, we simply wanted to add to it. It was a matter of answering a simple question in light of facts and figures recorded from patients at a national institutional level - a query frequently ignored and shrugged aside: do statins cause abnormal LFTs?

## Materials and methods

We conducted a retrospective observational cohort. It was held at the National Institute of Cardiovascular Diseases (NICVD), Karachi. All data were collected from the electronic database of NICVD for patients admitted from July 1, 2018, to December 31, 2018. Further data on follow-up were recorded from the same database at induction and at six months and 12 months post-induction. All labs were carried out free of cost for the patients. Patient confidentiality was ensured at all times.

The consecutive and non-probability sampling technique was used in the enrollment of patients in this study. Patients of either gender, aged ≥18 years that were admitted to the NICVD during the study period, were eligible for induction. Only patients already using a statin at any dose for at least a period of six months were considered for induction into the study. Liver function tests were recorded at induction and at six and 12 months post-induction. 

Meticulous care was taken to rule out other pathologies affecting liver function tests. Therefore, patients with fatty liver disease, alcoholic liver disease, congestive heart failure, chronic hepatitis, acute hepatitis, metabolic liver diseases, metabolic bone disease, chronic kidney disease, thyroid and parathyroid dysfunction, inherited hypercoagulable states, soft organ malignancy, metastatic cancer, and terminally ill patients were excluded from the study. Patients on chemotherapy, radiotherapy, or using herbal medications were also left out. Smokers and patients using tobacco products were also excluded.

Statistical analysis

Data were analyzed using the Statistical Package for the Social Sciences (SPSS) version 21.0 (IBM Corp., Armonk, NY). The mean and standard deviation was calculated for age, duration of statin use, and liver function tests. Frequency was calculated for the type of statin used, provisional diagnoses, and comorbids. A p-value of <0.05 was taken as significant.

## Results

Two hundred and four patients were eventually inducted into the study after undergoing extensive testing. The mean age of our study cohort was 53.41±10.13 years. The distribution ratio of females to males in this study was 1:4. The most common provisional diagnosis on admission was non-ST-elevation myocardial infarction (NSTEMI) affecting just over half of the patients. Concordantly, the most common comorbidity was hypertension, which was present in over three-quarters of the patients. General characteristics are summarized in Table 1.

**Table 1:** General characteristics of the patients inducted into the study NSTEMI=Non-ST-elevation myocardial infarction, AWMI=Anterior wall myocardial infarction, IWMI=Inferior wall myocardial infarction, ACS=Acute coronary syndrome, HTN=Hypertension, DM=Diabetes mellitus *Mortality after induction into the study after an adverse event (reason for admission)

	N=204
Age (mean)	53.41±10.13 years
Gender	
Male	160 (78.43%)
Female	44 (21.56%)
Diagnosis (reason for admission)	
NSTEMI	112 (54.90%)
AWMI	48 (23.52%)
IWMI	24 (11.70%)
LWMI	16 (7.84%)
ACS	4 (1.96%)
Comorbids	
HTN	144 (52.94%)
HTN + DM	28 (13.72%)
None	20 (9.80%)
DM	12 (5.88%)
Post induction mortality at one year*	Nil

The mean and median duration of statin use before induction into the study were 19.92±14.34 and 15 months, respectively. Combined with the follow-up period of one year, the duration of statin use in this study for most patients was approximately three years; a fairly large time span to analyze the effects of a given drug class on the liver and liver function tests.

One-hundred and seventy-six (86.27%) patients were already using rosuvastatin 20 mg/day at induction into the study; the remainders of the patients were using atorvastatin 40 mg/day. Other statins, such as fluvastatin and simvastatin, were seen during the study, however, their numbers were far too low to perform any significant statistical analysis and were left out. The types and duration of statin use are shown in Table 2.

**Table 2:** Duration and types of statins used in the study *indicates the time period a statin was being used before induction into the study **other doses of these statins were also recorded but their numbers were too low to perform any significant analysis, therefore, they were eventually not considered

	N=204
Duration of statin use* (mean)	19.92±14.34 months
Type and dose of statin used	
Rosuvastatin 20 mg/day**	176 (86.27%)
Atorvastatin 40 mg/day**	28 (13.72%)

The mean for alanine transaminase (ALT), aspartate transaminase (AST), and gamma-glutamyl transpeptidase (GGT) were all slightly raised at induction when adjusted for regional ranges [11]. All elevations, however, were less than 2x the upper limit of normal (ULN) for the specific enzyme; without exception, all LFTs demonstrated borderline elevation according to the latest guidelines [11]. This trend continued at six and 12 months post-induction as well. All abnormalities within the LFTs over time were found to be statistically insignificant. The results of LFTs with respect to statin use over time are summarized in Table 3.

**Table 3:** Liver function tests over time with respect to all statin use ALT= Alanine transaminase, AST=Aspartate transaminase, GGT=Gamma-glutamyl transpeptidase, ALP=Alkaline phosphatase, ACG=American College of Gastroenterology *Ranges have been adjusted for the local population as per ACG guidelines Source: [13]

	At inclusion	6 months	12 months	Normal ranges*	p-value
ALT (IU/L)	48.13±14.35	48.85±12.79	49.18±10.92	0-41	>0.05
AST (IU/L)	46.37±12.75	46.12±13.22	47.07±14.51	10-50	>0.05
GGT (IU/L)	37.21±15.51	37.59±12.48	38.51±16.54	0-37	>0.05
ALP (IU/L)	76.49±39.90	75.12±52.96	77.00±76.73	29-310 (adults)	>0.05

Patients using atorvastatin 40 mg/day demonstrated greater derangements of LFTs over time as compared to rosuvastatin 20 mg/day. All derangements in the LFTs were less than 2x the ULN. AST levels with atorvastatin showed continued elevation at six months and 12 months post-induction. ALT levels for both drugs mildly improved over the follow-up period and AST levels with rosuvastatin use also demonstrated mild improvement at the six and 12-month follow-up. No elevation or improvement in LFTs over time was found to be statistically significant. LFTs with respect to specific stain use over time are summarized in Table 4.

**Table 4:** Liver function tests over time with respect to specific statins ALT=Alanine transaminase, AST=Aspartate transaminase, GGT=Gamma-glutamyl transpeptidase, ALP=Alkaline phosphatase, ACG=American College of Gastroenterology *Ranges have been adjusted for the local population as per ACG guidelines Source: [13]

	At inclusion	6 months	12 months	Normal ranges*	p-value
Rosuvastatin 20 mg/day					
ALT (IU/L)	46.90±12.98	49.36±11.46	48.24±11.48	0-41	>0.05
AST (IU/L)	44.27±11.36	43.68±11.61	43.01±15.24	10-50	>0.05
Atorvastatin 40 mg/day					
ALT (IU/L)	55.85±20.71	51.22±16.07	48.40±9.23	0-41	>0.05
AST (IU/L)	59.57±13.95	59.37±19.84	62.01±19.33	10-50	>0.05

## Discussion

According to the latest guidelines issued by the American College of Gastroenterology (ACG), elevations of LFTs <2x ULN are considered borderline [13-14]. Once deleterious causes, such as chronic viral hepatitis, alcoholism, hemochromatosis, and autoimmune pathologies, have been ruled out, further evaluation is not required if a cause is known as the case was in our study.

Borderline elevations are almost always benign. These require no further intervention, and patients can be safely prescribed medications over long periods of time. The median time period of statin use before induction in our study was 15 months. Post induction, patients were followed up for another year. At no point during this period (approximately 3 years) were the LFTs elevated more than 2x ULN for either of the drugs analyzed.

ALT levels were slightly more elevated than AST levels in our study; this simply corresponds to the fact that ALT is a more specific marker of liver injury than AST [11]; for most patients, AST levels were below the reference range adjusted for the local population. All elevations were found to be statistically insignificant. As mentioned previously, these borderline elevations do not merit further investigation or intervention and, therefore, statins can be used without hesitation.

The Greek Atorvastatin and Coronary Heart Disease Evaluation (GREACE) study established the safety and efficacy of statin use in cardiovascular diseases with abnormal liver function tests [15]. Many results from our study mimic those of the GREACE ad-hoc analysis. Improved cardiovascular outcomes, no evidence of clinically significant liver injury, or decompensation over time and only mild elevations of LFTs with statin use are a few of them. While the GREACE analysis worked on atorvastatin mainly, in our study, these outcomes were seen with rosuvastatin as well.

There were two major differences between our study and the GREACE analysis. One, we did not induct patients with fatty liver disease in our study, whereas a good proportion of patients in the GREACE analysis had it. Secondly, all of our patients were using statin before induction into the study and all patients had some elevations of LFTs, this was not the case with the GREACE analysis. These were due to differing study protocols and were statically and clinically insignificant. 

Studies previously analyzing the effects of statins in fatty liver disease and cardiovascular disease [15-16] have recorded safety and improvement in LFTs over time along with improved cardiovascular outcomes. Similar improvements in LFTs with atorvastatin use were seen in the GREACE study. It would stand to reason that patients without fatty liver disease would exhibit similar benefits. However, the evidence for this was lacking in our study.

There were cases where ALT and/or AST levels decreased over the follow-up period, but such occurrences were far few and between. In the vast majority of the patients, both ALT and AST were mildly elevated even before induction. These levels either remained steady or increased mildly over the follow-up period. These elevations did not have any clinical or statistical impact eventually but the return of LFTs to optimal levels was not the norm. This is the one instance where our study differs markedly from previous data.

Previous studies and authors have argued against the regular monitoring of LFTs for a multitude of reasons, ranging from economic burden to clinical insignificance [17-18]. The authors of this study would, however, like to side with caution and advice reasonable intervals for liver function testing in patients using statins. Factors such as cost to the health care system, patient feasibility and ease, quality of testing facilities, drug compliance, and comorbids should all be considered in determining these intervals.

Our opinion is driven by a single observation that a very small percentage of patients in our study did develop moderate elevations of LFTs (>5x ULN). These patients are prime candidates for the development of DILI. Such patients require regular testing and stoppage of the culprit drug (if identified) should LFTs remain elevated chronically, as DILI can cause irreversible liver damage. There is no other method to identify these patients except to carry out regular LFTs. In a country with high rates of liver cirrhosis [19], we rather stay safe than sorry.

Our results indicate that statins do cause borderline elevation of LFTs overtime. These abnormalities are dose dependant; patients using atorvastatin 40 mg/day had greater elevations for both ALT and AST as compared to rosuvastatin 20 mg/day. Glueck CJ et al. previously demonstrated little to no derangement of LFTs with rosuvastatin 5-10 mg/day [20]. Kasliwal R et al. recorded more frequent and higher elevations of LFTs with the use of rosuvastatin 40 mg/day as compared with rosuvastatin 10 mg/day [21]. Shepherd J et al. presented a similar trend with rosuvastatin use [22].

So yes, there is a correlation between abnormal LFTs and statin use. But more importantly, in light of all the previous data discussed here and our results, it is imperative to remember that these minor derangements do not carry any clinical significance. The pros of treating and improving cardiovascular outcomes far outweigh the con of minor LFTs derangement; previous data, as well as ours, show that over long periods of time, there is no liver damage of any clinical significance physiologically or anatomically [22]. In reality, many of these studies attributed deranged LFTs to other causes such as renal compromise and fatty liver disease. We have tried our level best to rule out all other such pathologies.

Limitations of the study

The scope of this study was limited by a number of factors. Most notably, we were not able to analyze the effect of other drugs that cardiac patients inevitably use with statins on LFTs. Neither were we able to assess any drug interactions that might have taken place. The meticulous workup that was done to rule out different causes of liver disease was only carried out once, with the exception of the ultrasound abdomen. The same exclusion process reduced our sample size; therefore, we could not record data for other statins. Nutritional, caloric, and lifestyle assessments were not done.

## Conclusions

There is a correlation between deranged LFTs and statin use. This correlation is dose-dependant but is clinically and statistically insignificant. It should not deter physicians from prescribing statins nor should it raise any red flags in cardiac patients already taking them.
